# Leisure time physical activity in a 22-year follow-up among Finnish adults

**DOI:** 10.1186/1479-5868-9-121

**Published:** 2012-10-02

**Authors:** Katja Borodulin, Tomi E Mäkinen, Päivi Leino-Arjas, Tuija H Tammelin, Markku Heliövaara, Tuija Martelin, Laura Kestilä, Ritva Prättälä

**Affiliations:** 1 National Institute for Health and Welfare, PO Box 30, Helsinki, FI-00271, Finland; 2 Finnish Institute of Occupational Health, Helsinki, Finland; 3 LIKES Research Center for Sport and Health Sciences, Jyväskylä, Finland; 4 The Finnish Youth Research Network (FYRN), Helsinki, Finland

**Keywords:** Exercise, Health behavior, Occupation, Prospective studies, Socioeconomic position

## Abstract

**Background:**

The aim of this study was to explore long-term predictors of leisure time physical activity in the general population.

**Methods:**

This study comprised 718 men and women who participated in the national Mini-Finland Health Survey from 1978–1980 and were re-examined in 2001. Participants were aged 30–80 at baseline. Measurements included interviews, health examinations, and self-administered questionnaires, with information on socioeconomic position, occupational and leisure time physical activity, physical fitness, body mass index, smoking, alcohol consumption, and physical functional capacity. Analyses included persons who were working and had no limitations in functional capacity at baseline.

**Results:**

The strongest predictor of being physically active at the follow-up was participation in physical activity at baseline, with an OR 13.82 (95%CI 5.50-34.70) for 3 or more types of regular activity, OR 2.33 (95%CI 1.22-4.47) for 1–2 types of regular activity, and OR 3.26 (95%CI 2.07-5.15) for irregular activity, as compared to no activity. Other determinants for being physically active were moving upwards in occupational status, a high level of baseline occupational physical activity and remaining healthy weight during the follow-up.

**Conclusions:**

To prevent physical inactivity among older adults, it is important to promote physical activity already in young adulthood and in middle age and to emphasize the importance of participating in many types of physical activity.

## Introduction

As the health benefits of physical activity are widely reported [1,2], it is important to study why people choose to be physically active and which factors predict engagement in physical activity in a follow-up design. Previous cross-sectional studies [3-6] and reviews [7,8] suggest the following factors are associated with higher levels of leisure time physical activity: younger age, being married, not having small children, higher socioeconomic position, lower body mass index (BMI), low occupational physical activity, good functional capacity, and other health behaviors such a being a non-smoker. Nevertheless, less is known on the long term predictors of physical activity, particularly among adults or older adults. Previous prospective studies have reported that leisure time physical activity fluctuates over the life course and that tracking of activity is low and heterogeneous from childhood to adulthood or during adulthood [8-13]. As for other predictors, three studies suggest that adults in a lower socioeconomic position may decrease their activity more than those in a higher socioeconomic position, [14-16] while another study found weak or no systematic differences in physical activity across socioeconomic position over time [11]. Furthermore, retirement is reported to be associated with an increase in physical activity [11,17] and having children with a decrease in activity levels [11].

The current literature lacks prospective population studies among adults [7]. It would be important to know what predicts participation in physical activity over a longer time. To address the gap in the current literature, the aim of this study was to explore predictors of leisure time physical activity in a 22-year follow-up using a population based sample of Finnish adults.

## Methods

The baseline measurements were part of the national Mini-Finland Health Survey carried out from 1978–1980, in which a population-based two-stage stratified cluster sample of Finns aged 30–80 years were invited to participate in a health examination [18]. The follow-up examination was implemented 22 years later as part of the Health 2000 Survey [19]. The sample comprised 1278 participants who were alive in 2000 and were living in the regions of seven large cities of Finland; Helsinki, Kuopio, Lahti, Oulu, Salo, Tampere, and Turku. Participation rate at the follow-up was 70% (n = 892). Participants who were working at baseline, had no limitations in their functional capacity at baseline, and had no missing information on any of the used variables, were included, leaving 718 men and women in the cohort for the analyses. The survey was approved by the Ethical Committee for Epidemiology and Public health in the hospital district of Helsinki and Uusimaa, Finland. All participants gave written informed consent.

Measurements included interviews by trained public health nurses, health examinations by physicians, and self-administered questionnaires. At the baseline and follow-up, participants had their height and weight measured for a calculation of BMI (kg/m^2^) Measurements of BMI, smoking, and occupational status were carried out similarly at baseline and follow-up, while some alterations were done to questions on leisure time physical activity, alcohol use and education.

Leisure time physical activity at the baseline was queried with a question: “How much do you move about and how hard do you exert yourself physically in your spare time?”, with the response options 1) only a little physical exercise, 2) physical exercise in connection with other hobbies or irregularly, and 3) regular physical exercise. For regular exercise, the participant was asked to list the most common types of activity he or she engaged in. In the analyses, the first two response options were treated as “no activity” and “irregular activity”, respectively, and the third group, regular activity group, was divided into those reporting “1-2 types of regular activity” and “3 or more types of regular activity”.

The level of leisure time physical activity at the follow-up was the main outcome variable in the analyses and was slightly modified from the baseline: “How much do you exercise and strain yourself physically in your leisure time?” The response options were: 1) In my leisure time I read, watch TV and do other activities in which I do not move much and do not strain me physically; 2) In my leisure time I walk, cycle, and move in other ways at least 4 hours per week (moderate intensity); 3) In my leisure time I exercise at least 3 hours per week (vigorous intensity); and 4) In my leisure time I practice regularly several times per week for competition (very vigorous intensity). Response option 1) was treated as the ‘inactive’ category and the other categories were merged into a ‘physically active’ category. This instrument has shown good internal validity against all-cause and cardiovascular mortality [20].

Occupational physical activity was assessed at baseline with the question: “How much do you move about at work and how strenuous is your work physically?” The six response options from light sedentary work to very heavy manual work were categorized into low, middle, and high. A question on self-rated physical fitness level at baseline was formulated: “How good do you consider your physical fitness?” with the response options: very good, fairly good, fair, quite poor, and very poor. The ‘quite’ and ‘very poor’ categories were combined in the analyses due to low frequencies.

Information on education was obtained at baseline and follow-up from the questionnaire and was categorized as low (primary level), middle (secondary level), and high (tertiary level) education for the analyses. Current or the most recent occupation was given as an open answer and was further categorized into blue-collar, lower white-collar, and upper white-collar workers. Smoking status and alcohol consumption were self-reported at both measurement points. Alcohol consumption was determined as the amount of alcohol in grams per month and was divided into gender-specific thirds.

For the analyses, new variables were created, based on the combined information from both the baseline and follow-up. The new variables and their categories were the following: educational status over 22 years (remained as highly educated; and no change or change towards higher or lower educational level i.e. later referred to as other categories), occupational status over 22 years (remained as upper white-collar; remained as lower white-collar; remained as blue-collar; moved upwards; and moved downwards), change in BMI over 22 years (healthy weight-healthy weight both at baseline and follow-up respectively; overweight-overweight; overweight-healthy weight; and healthy weight-overweight), smoking status over 22 years (non-smoker-non-smoker; smoker-smoker; and changed smoking behavior), and alcohol consumption over 22 years (low-low; changed consumption; and high-high). The cell sizes were too small for some categories, such as those whose weight status had changed from overweight to healthy weight (n = 17), forcing us to merge some categories.

### Statistical methods

Logistic regression analysis was the main statistical method. The likelihood of being physically active during leisure time at follow-up was examined using odds ratios (OR) with 95% confidence intervals (95% CI). First, age and gender adjusted logistic regression models were carried out for each factor separately. Then, the fully adjusted model was implemented and included all factors. Men and women were pooled together in the analyses. Interaction tests of age and gender for all associations between leisure time physical activity and independent variables were implemented and reported where statistically significant interactions were found. Hosmer-Lemeshow goodness-of-fit statistics were tested, showing adequate fit in the adjusted models. Statistical analyses were carried out using SAS software program (version 9.1., Cary, NC).

## Results

At the follow-up study, the Mini-Finland cohort (n = 719) with a mean age of 62.5 years (range 50–94 years) and 45% of them were men, and 34% were working full-time. During the follow-up, 20% remained in the upper white-collar class and 20% in the blue-collar class, while 6% moved upwards in occupational status. For BMI, 28% remained a healthy weight, 36% remained overweight, and 36% had a change in weight.

The predictors of regular moderate to vigorous intensity leisure time physical activity after a 22-year follow-up were explored among the cohort of Mini-Finland participants. In the age and gender adjusted models (Table 1), leisure time physical activity was more common among those who moved upwards in occupational status during the follow-up, had high occupational physical activity at baseline, reported higher leisure time physical activity at baseline, had good self-rated fitness at baseline, had remained healthy weight or changed weight status during the follow-up, were non-smokers at baseline and follow-up and remained a low alcohol consumer or changed alcohol consumption during the follow-up.

**Table 1:** Predictors for being physically active in leisure time (LTPA) in the 22-year-follow-up

	**Total**	**Physically active**	**OR for LTPA**^**a**^	**95% CI**	**Adjusted OR for LTPA**^**b**^	**95% CI**
	**n**	**n (%)**				
Educational status over 22 years						
Remained as highly educated	123	94 (76.4)	1.00	-	1.00	-
Other categories	595	467 (78.5)	1.14	0.72-1.82	1.01	0.50-2.04
Occupational status over 22 years						
Remained as blue-collar	140	114 (81.4)	1.00	-	1.00	-
Remained as lower white-collar	221	168 (76.0)	0.71	0.40-1.26	1.03	0.51-2.08
Remained as upper white-collar	147	110 (74.8)	0.67	0.38-1.46	0.80	0.34-1.88
Moved upwards	42	40 (95.2)	4.55	1.03-20.14	5.52	1.16-26.35
Moved downwards	168	129 (76.8)	0.76	0.43-1.36	1.00	0.50-2.00
Employment status at follow-up						
Retired, part time work, unemployed, other	473	376 (79.5)	1.00	-	1.00	-
Working fulltime	245	185 (75.5)	0.65	0.41-1.03	0.62	0.36-1.04
Baseline occupational physical activity						
High	83	73 (88.0)	1.00	-	1.00	-
Middle	331	262 (79.2)	0.51	0.25-1.06	0.40	0.17-0.95
Low	304	226 (74.3)	0.39	0.19-0.80	0.27	0.11-0.68
Baseline leisure time physical activity						
No activity	162	95 (58.6)	1.00	-	1.00	-
Irregular activity	341	278 (81.5)	3.20	2.10-4.87	3.26	2.07-5.15
1-2 types of regular activity	87	66 (75.7)	2.28	1.27-4.11	2.33	1.22-4.47
3 or more types of regular activity	128	122 (95.3)	14.49	6.02-34.88	13.82	5.50-34.70
Baseline self-rated physical fitness						
Poor or very poor	40	27 (67.5)	1.00	-	1.00	-
Average	270	202 (74.8)	1.45	0.71-2.96	0.90	0.41-2.01
Fairly good	248	193 (77.8)	1.70	0.82-3.52	0.94	0.41-2.13
Good	151	132 (87.4)	3.39	1.49-7.73	1.65	0.66-4.15
Change in body mass index over 22 years						
Remained overweight	262	182 (69.5)	1.00	-	1.00	-
Remained healthy weight	201	175 (87.1)	3.16	1.91-5.23	3.19	1.85-5.48
Change in body mass index	255	204 (80.0)	1.85	1.22-2.82	1.70	1.08-2.69
Smoking status over 22 years						
Remained as smoker	89	63 (70.8)	1.00	-	1.00	-
Remained as non-smoker	363	290 (79.9)	1.72	1.01-2.93	1.37	0.74-2.55
Changed smoking behavior	266	208 (78.2)	1.48	0.85-2.57	1.55	0.83-2.90
Alcohol consumption over 22 years						
Remained high	118	82 (69.5)	1.00	-	1.00	-
Remained low	250	202 (80.8)	1.99	1.18-3.34	1.49	0.82-2.73
Changed consumption	350	277 (79.1)	1.74	1.08-2.80	1.37	0.80-2.36

Participants of the Mini-Suomi cohort (n = 718) who were working and had no limitations in functional capacity at baseline.

^a^ Age and gender adjusted odds ratios (OR) with 95% confidence intervals (95% CI). Statistically significant interactions were found between employment status at follow-up and gender (p = 0.03).

^b^ Adjusted for age, gender, and all variables in the table.

In the fully adjusted model (Table 1), the strongest predictor of leisure time physical activity was participation in leisure time physical activity at baseline, with an OR of 13.82 (95%CI 5.50-34.70) for 3 or more types regular activity, OR of 2.33 (95%CI 1.22-4.47) for 1–2 types of regular activity, and OR of 3.26 (95%CI 2.07-5.15) for irregular activity, as compared to no activity at baseline. Other significant predictors were moving upwards in occupational status (OR 5.52, 95%CI 1.16-26.35) as compared to remaining as a blue-collar worker during the follow-up, low (OR 0.27, 95%CI 0.11-0.68) and middle (OR 0.40, 95%CI 0.17-0.95) level of baseline occupational physical activity as compared to high baseline occupational physical activity, remaining a healthy weight (OR 3.19, 95%CI 1.85-5.48) and having a change in weight status (OR 1.70, 95% CI 1.08-2.69) as compared to remaining overweight during the follow-up.

## Discussion

These population-based data among 718 working age and older adults suggest that the most important predictor of leisure time physical activity after a 22-year follow-up was the baseline leisure time physical activity. Participants who at baseline reported 3 or more types of leisure time physical activity were manifold more likely to report leisure time physical activity at the follow-up than inactive participants at the baseline. Other important predictors for being physically active during leisure time after 22 years were high occupational physical activity at baseline, moving upwards in occupational status during 22 years, and healthy BMI category at both baseline and follow-up.

Baseline physical activity strongly predicted participation in leisure time physical activity in later life in this study. Three prospective studies [11,13,15] suggested that physical activity level varies during adulthood and that tracking is low. However, a 28-year follow-up study among metal workers [13] reported that baseline physical activity was the strongest predictor of later physical activity.

Only a few studies have used large or population-based samples and have included predictors of physical activity [11,13-15,21]. We found that those participants who moved upwards in their occupational status were 5.5 times more likely to report leisure time physical activity after 22 years as compared to those who remained blue-collar workers. Previous studies have reported lower socioeconomic status as predicting later leisure time physical activity, but none of them reported on the potential change in occupational status [14-16,21]. One study indicated that an increase in education predicted increased leisure time physical activity after 2 years follow-up [22]. This and our study suggest that health behaviors may change during the life course with a change in a socioeconomic position. One logical explanation could be that a higher occupational position brings material means to make choices on physical activity. Another plausible explanation could be that those who move upwards in their socioeconomic position adopt new social norms, e.g. at work and in personal life. A new work place, colleagues, and friends give the needed social support and role model to be physically active during leisure time.

The effect of occupational physical activity on later leisure time physical activity has not been reported before in population studies. Interestingly, we found that higher occupational physical activity at baseline predicted participation in leisure time physical activity. This could be explained by the healthy worker effect, i.e. those who are fit and physically capable of carrying out their physically demanding job tasks [23] are a selected population as they survive and remain in the sample during the follow-up.

Two studies [13,14] included BMI in their analyses, but BMI had no statistical significance. Another study, although with just a 2-year follow-up, reported that gaining weight was associated with decreased leisure time physical activity [22]. In the present cohort, it is suggested that people who remained in a healthy weight range at baseline and follow-up are also physically active after 22 years. It is not possible to determine from these data the extent to which physical activity contributed to achieving a healthy weight status at follow-up.

Previously, it has been suggested [13,14,21] that age or gender may predict later physical activity, but in the present analyses neither age nor gender were significant factors. It has also been suggested [21] that sociodemographic factors may play an important role in predicting long term physical activity, which was seen this study.

### Methodological issues

The baseline Mini-Finland sample was a cross-sectional representative sample of Finns aged 30 years and above, with an excellent participation rate of 90%. The follow-up population, however, suffered from selection bias, as some of the original cohort members had died and only those living in large municipalities were invited to participate. We know that those from lower socioeconomic position are less likely to participate in population-based studies and have a higher risk of premature death than those from a high socioeconomic position [15,24,25], which was also the case in our study. Furthermore, from those who were invited to the re-examination, those more likely to participate were from the younger age cohorts, upper-white collar workers, higher educated, as well as those with a higher level of leisure time physical activity at baseline and a healthy BMI at baseline. Also, those who had a less demanding physical work-load at baseline occupation were more likely to take part in the re-examination. Thus, our cohort is not a representative sample of the Finnish population in the follow-up and our findings are not generalizable to the entire population.

Another limitation of this study is the measurement of leisure time physical activity, which was based on self-reported weekly amount of activity and was slightly altered between the two time points. At baseline, more activity categories were possible than at the follow-up, allowing the identification of regular exercisers from lower levels of exercise and physical inactivity. For the question at follow-up, only two physical activity categories were created; the inactive and active categories. The physical activity measurements at both time points are crude and do not allow proper measurement of frequency, intensity, or duration. It should be born in mind that the questions originate from population studies in the 1960s and at that time physical activity epidemiology was yet to develop. Even though our cut-off points for active and inactive groups do not follow the current recommendations for physical activity, we believe our instrument categorizes the two activity groups well. The likelihood for misclassification is low. Those participants who were categorized as inactive at the follow-up, are a real target group for health promotion.

In some of the statistical models, the confidence intervals were large; for example for the highest physical activity category in the baseline, 95% confidence interval was from 5.50 to 34.70 (OR 13.82, as compared to the reference group of inactivity). A wide confidence interval reflects poor precision for the statistical estimate, in this case suggesting large variation in the estimates between the categories of leisure time physical activity. This could have been corrected by organizing the response categories differently, but we would have lost some of the interesting results. By this we mean that reporting three of more types of regular activity increased the likelihood of reporting leisure time physical activity after 22 years.

## Conclusions

Earlier participation in leisure time physical activity was the most important predictor of later leisure time physical activity. To prevent physical inactivity among older adults, it is important to promote physical activity already in young adulthood and middle age. Participation in a larger variety of activities should also be emphasized when promoting physical activity at population level. In addition, as people move upwards in their occupational status, they are likely to increase their activity level. The associations between health behaviors during the life course should be studied in more detail using a socioeconomic approach.

## Competing interest

The authors declare that they have no competing interests.

## Authors’ contributions

KB was the leading author and RP the director of the research group. MH, LK, and TM were the experts of the two datasets that were collected during the 22 years and were essentially involved in the implementation and management phases of the data collections. TEM, PL-A, THT have coauthored and provided their expertise in their own fields, particularly in exercise science, occupational health, and socioeconomic variation in physical activity. All the authors have substantially contributed to the planning and writing of the manuscript. All authors read and approved the final manuscript.
